# Meta-analysis for dorsally displaced distal radius fracture fixation: volar locking plate versus percutaneous Kirschner wires

**DOI:** 10.1186/s13018-015-0252-2

**Published:** 2015-07-15

**Authors:** Shuang-Le Zong, Shi-Lian Kan, Li-Xin Su, Bin Wang

**Affiliations:** Department of Orthopedics Institute, Tianjin Medical University, Tianjin, 300070 People’s Republic of China; Department of Orthopedics Institute, The Second Hospital of Tangshan, Tangshan, 063000 Hebei Province People’s Republic of China

**Keywords:** Distal radius fracture, K-wire, Volar locking plate, Meta-analysis

## Abstract

**Background:**

Dorsally displaced distal radius fractures (DDDRF) are frequent injuries in clinical practice. Traditional percutaneous Kirschner wires (K-wire) and open reduction with volar locking plate (VLP) are the two most common surgical fixation techniques used to manage DDDRF. However, there is no current consensual evidence to guide the selection of one technique over the other. Therefore, we undertook a systematic search and meta-analysis to compare clinical outcomes and complications of these two treatment approaches for DDDRF.

**Methods:**

The following electronic databases were searched by two independent reviewers, up to April 2015: PubMed, ScienceDirect and Wiley Online Library. High-quality randomized controlled trials (RCTs) comparing VLP and percutaneous K-wire fixation for DDDRF were identified. Pooled mean differences were calculated for the following continuous outcome variables: disabilities of the arm, shoulder and hand (DASH) score, grip strength and wrist range of motion. Pooled odds ratios were calculated for rates of total postoperative complications, including superficial infection, deep infection, complex regional pain syndrome (CRPS), carpal tunnel syndrome (CTS), neurological injury, tendon rupture, tenosynovitis, loss of reduction and additional surgery to remove hardware. The meta-analysis was completed using RevMan 5.3 software.

**Results:**

Seven RCTs, with a total of 875 patients, were included in our meta-analysis. Open reduction internal fixation (ORIF) with VLP fixation provided statistically lower DASH scores, reduced the incidence of total postoperative complications and specifically lowered the rate of superficial infection, when compared, over a 1-year follow-up, to percutaneous K-wire fixation. VLP fixation also provided significantly better grip strength and range of wrist flexion and supination in the early 6-month postoperative period, compared with percutaneous K-wire fixation.

**Conclusion:**

ORIF with VLP fixation provided lower DASH scores and reduced total postoperative complications, most specifically lowering the risk for postoperative superficial infection compared to K-wire fixation over a 1-year follow-up period. However, superficial pin track infections do not cause clinical debility in the vast majority of cases. Thus, the claim of reduced superficial infection rate may not be clinically important. The only reasonable conclusion that can be drawn is that at present, there is insufficient data even on our meta-analysis to help the clinician make an informed choice.

## Background

Distal fractures of the radius comprise the largest portion of orthopaedic fractures, accounting for one sixth to one fourth of all fractures treated in clinical emergency departments [1]. These fractures are more common in postmenopausal women. The lifetime risk of sustaining a fracture of the distal radius is 15 % for women and 2 % for men [2]. Among all fractures, dorsally displaced distal radius fractures (DDDRF) are the most common. As the population is ageing, the specific incidence of this fracture type will, undoubtedly, increase in the coming years. In the past, many of these fractures were managed nonoperatively. However, the high incidence of malunion, associated with nonoperative management led to poor clinical outcomes, including pain and disability. Advances in internal fixation techniques have resulted in increased reliance on operative approaches for the management of DDDRF. Closed reduction and fixation with percutaneous Kirschner wires (K-wire) has historically been the most common operative approach for distal radius fractures, providing a relatively quick and inexpensive treatment method [3]. It was accepted that percutaneous pinning with K-wires should be considered for patients with unstable extra-articular or simple intra-articular distal radius fractures [4]. However, percutaneous K-wires are not load-bearing devices and, therefore, cannot protect against radial shortening, especially in osteoporotic bone, which has been associated with poor postoperative functional outcomes [5]. Open reduction and internal fixation using a volar locking plate (VLP) is increasingly being used as an alternative to K-wire fixation, providing stability and allowing early mobilization of the hand and wrist [6]. Both the K-wire and VLP fixation techniques present different advantages and disadvantages.

While several prospective randomized controlled trials [7–13] and comparative trials [14–16] have been conducted to compare both the K-wire and VLP fixation techniques for the management of DDDRF, the optimal surgical management is still debated. In fact, there is little evidence in clinical practice to support one fixation technique over the other. To our knowledge, comparison of the VLP and K-wire fixation techniques has not been systematically evaluated through a meta-analysis. Therefore, we undertook a comprehensive meta-analysis using RCTs to evaluate the evidence for both VLP and K-wire fixation techniques in the treatment of DDDRF. Postoperative functional outcomes and complication rates were pooled from published trials comparing surgical and functional outcomes for VLP and K-wire fixations. We also performed a subgroup analysis to evaluate functional outcome at different periods of follow-up, with the aim of discovering the rehabilitation tendency based on the best available evidence.

## Methods

### Retrieve strategy

The meta-analysis was performed according to the guidelines from the Preferred Reporting Items for Systematic Reviews and Meta-Analyses (PRISMA) Statement for reporting from a wide range of systematic reviews [17]. The following electronic databases were searched by two independent reviewers, up to April 2015: PubMed, ScienceDirect and Wiley Online Library. High-quality RCTs, comparing VLP with K-wire fixation for the management of DDDRF, were selected. Only RCTs published in the English language were considered. Databases were searched using the following key words and combinations: (distal radius [Title/Abstract]) OR distal radial [Title/Abstract]) AND random*. The electronic search was complemented by a manual search of the reference lists of relevant articles.

### Selection criteria and quality assessment

The following inclusion criteria were applied for identification of relevant studies:

1. Target population: patients >18 years old, closed, unilateral, dorsally displaced distal radius fractures
2. Methodological criterion and intervention: all prospective RCTs and intervention studies comparing VLP with K-wire fixation for DDDRF
3. Outcome: reporting on at least one of the clinical outcomes of interest, namely rate of postoperative complications, clinical results, and radiological outcomes

The following exclusion criteria were applied:

1. Patients with bilateral fractures, multiple injuries, radiographic evidence of preexisting hand and wrist arthritis, dementia and open fractures
2. Trials with retrospective design, observational studies, case reports or review, biomechanical studies, and animal or cadaver studies.

According to the Cochrane handbook for systematic reviews of interventions 5.3, the methodological quality of included RCTs was independently assessed by two authors (Zong and Su), using a modified version of the generic evaluation tool used by the Cochrane Bone, Joint and Muscle Trauma Group. Disagreements between quality assessments were resolved by discussion. A third author (Kan) adjudicated the decision when no consensus could be achieved. Bias was evaluated, using the ‘Assessing the Risk of Bias’ table [18]. Bias was evaluated on the following key domains: random sequence generation, allocation concealment, blinding of participants and personnel, blind outcome assessment, incomplete outcome data, selective reporting and other sources of bias.

### Data extraction

Two of authors (Zong and Su) independently extracted relevant data from the retrieved articles, including study design, patient characteristics, surgical interventions and patient-based outcomes. The extracted data were re-examined by another author (Kan).

### Data synthesis and analysis

Meta-analysis was performed using the Review Manager (RevMan version 5.3, Copenhagen, Denmark, The Nordic Cochrane Centre) programme, including graphic representation of the pooled data. For dichotomous variables, odds ratios (OR) and 95 % confidence intervals (CI) were calculated. For continuous data, means and standard deviations were used to calculate weighted mean differences (WMD), including 95 % CI. Statistical heterogeneity between studies was formally tested with the standard chi-square test to assess inconsistency in a study’s results. The *I*^2^ value was calculated to estimate the size of the heterogeneity. Regardless of statistical heterogeneity, we pooled all results using a random-effect model, with the level of significance set at *p* = 0.05. A probability of *p* < 0.05 was regarded to be statistically significant. We also assessed the presence of bias within the published data by using a funnel plot of the reported primary outcomes.

## Results

### Study characteristics

**Fig. 1:**
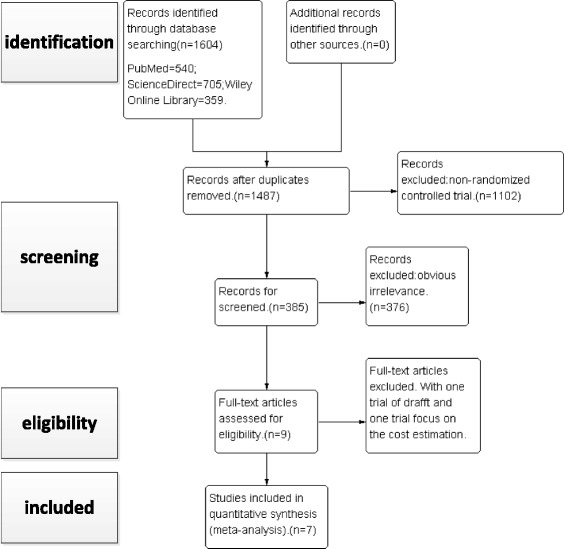
Flow chart summarizing the selection process of RCTs. *RCT*, randomized control trials

**Fig. 2:**
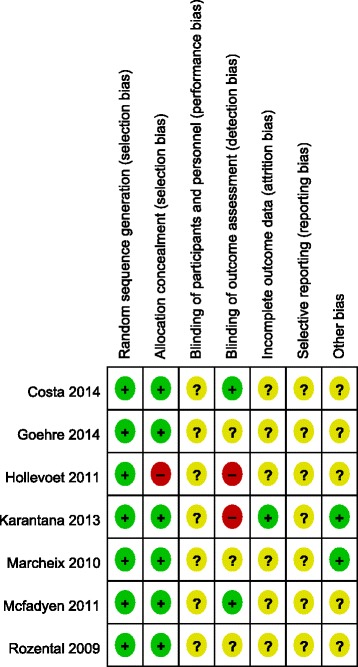
Methodological quality of included RCTs. This ‘risk of bias’ tool incorporates assessment of randomization (sequence generation and allocation concealment), blinding (participants, personnel and outcome assessors), completeness of outcome data, selection of outcomes reported and other sources of bias. The items are scored a ‘yes’, ‘no’, or ‘unsure’. *RCT*, randomized control trials

**Fig. 3:**
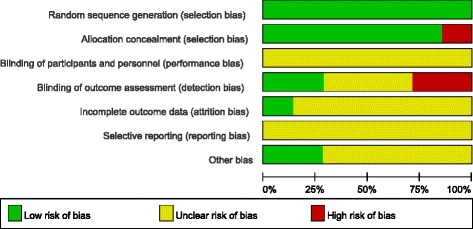
Risk of bias. Each item of the ‘risk of bias’ assessment is shown as a percentage across all included randomized control trials, indicating the proportion of different levels of risk of bias for each item

Our search strategy is shown in Fig. 1. Our search identified a total of 1604 citations as potentially relevant to our meta-analysis. Identified citations were screened by their title and abstract and complete articles read as required. Seven studies [7–13] satisfied our inclusion criteria and were entered into the meta-analysis. These seven RCTs provided complete data sets, at all time points of measurement in the trial, for a total of 875 patients. Relevant characteristics of patients included in the meta-analysis are reported in Table 1. While the overall quality of the RCTs was high, certain methodological limitations were identified and are shown in Figs. 2 and 3.

**Table 1:** Characteristics of the six included studies in the meta-analysis

Study (author, year)	Rozental 2009		Marcheix 2010		Hollevoet 2011		Mcfadyen 2011		Karantana 2013		Costa 2014		Goehre 2014	
Design	RCT		RCT		RCT		RCT		RCT		RCT		RCT	
Treatment	VLP	Pin	VLP	Pin	VLP	Pin	VLP	Pin	VLP	Pin	VLP	Pin	VLP	Pin
Sample number	23	22	50	53	20	20	27	29	66	64	231	230	21	19
Age (years)	51(19 to 77)	52(24 to 79)	75 ± 11	73 ± 11	>50		26–80	18–80	18 to 73		58.3 ± 14.9	59.7 ± 16.4	71.3 ± 5.7	73.8 ± 8.9
Male/female	7/16	4/17	12/38	5/48	NA		12/15	11/18	NA		NA		3/18	0/19
AO classification (A/B/C)	10/0/13	6/0/15	17/0/33	23/0/29	NA		27/0/0	29/0/0	27/0/39	28/0/36	149/5/75	157/3/66	18/0/3	15/0/4
Follow-up (month)	12		6		12		6		12		12		12	

*RCT* randomized controlled trial, *VLP* volar locking plate, *NA* not available

### Meta-analysis results

#### Mean difference in DASH scores

**Fig. 4:**
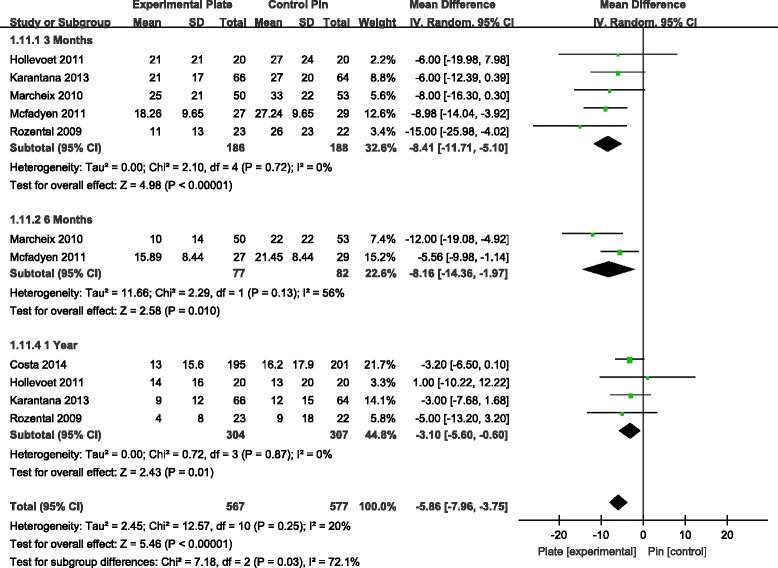
Forest plot illustrating the meta-analysis of the DASH score at 3, 6 and 12 months postoperatively. *DASH*, disabilities of arm, shoulder and hand

The DASH score was one of the most commonly reported functional outcome measures among the seven studies [7–13]. Analysis of the pooled DASH score data revealed a significant difference in score favouring the VLP over the K-wire fixation technique at 3, 6 and 12 months, postoperatively (Fig. 4). Over a 1-year postoperative period, patients who had been managed with VLP fixation had significantly lower DASH scores compared to scores for patients who had been managed with percutaneous K-wire fixation. This difference in score was specifically apparent over the first three postoperative months, followed by a stable trend over the remaining assessment time points.

#### Mean difference in grip strength and wrist ROM

**Fig. 5:**
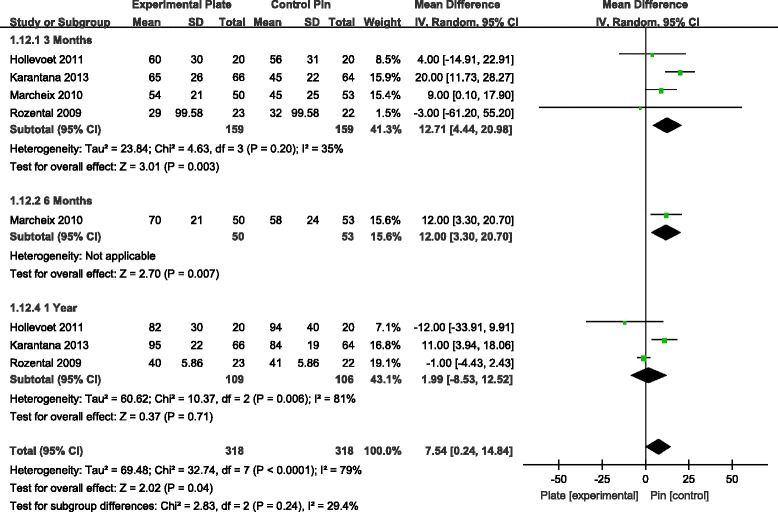
WMD estimates for grip strength 3, 6 and 12 months postoperatively. *WMD*, weighted mean difference

**Fig. 6:**
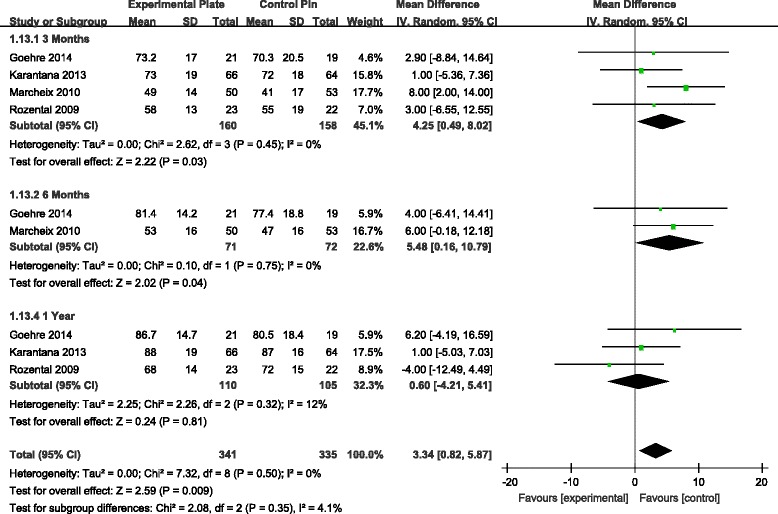
WMD estimates for range of wrist flexion at 3, 6 and 12 months postoperatively. *WMD*, weighted mean difference

**Fig. 7:**
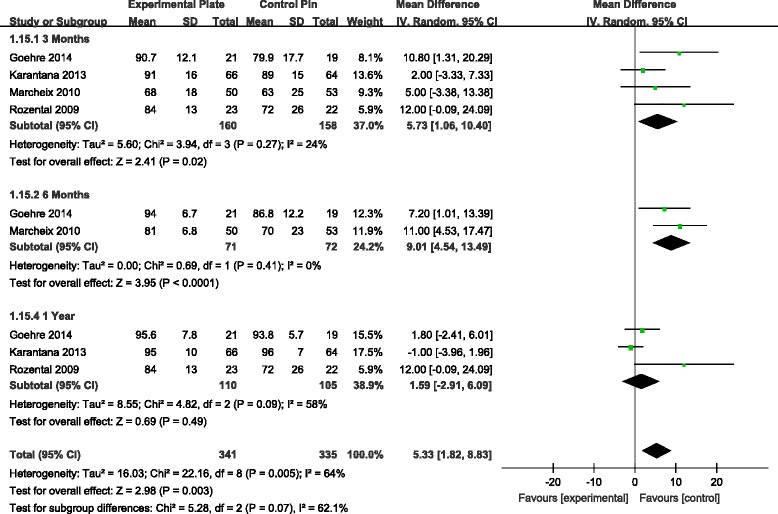
WMD estimates for range of wrist supination at 3, 6 and 12 months postoperatively. *WMD*, weighted mean difference

Grip strength was documented in four studies [9–11, 13]. Analysis revealed a significant difference in grip strength favouring VLP fixation at 3 and 6 months, but not at 1 year, postoperatively (Fig. 5). For wrist range of motion (ROM), there was a statistically significant difference in the range of wrist flexion and supination, with higher ROM values associated to the VLP fixation technique at 3 and 6 months, but not at 1 year postoperatively (Figs. 6 and 7). There were no other significant effects of fixation techniques on the other wrist ROMs (Table 2).

**Table 2:** Comparisons of clinical results postoperatively at 3, 6 and 12 months

Outcomes	Study numbers	Participants		WMD	95% CI	*p* value
		VLP	Pin			
Grip strength						
3 months	4	159	159	12.71	[4.44, 20.98]	0.003*
6 months	1	50	53	12	[3.30, 20.70]	0.007*
1 year	3	109	106	1.99	[−8.53, 12.52]	0.71
Flexion						
3 months	4	160	158	4.25	[0.49, 8.02]	0.03*
6 months	2	71	72	5.48	[0.16, 10.79]	0.04*
1 year	3	110	105	0.60	[−4.21, 5.41]	0.81
Extention						
3 months	4	160	158	3.02	[−0.86, 6.91]	0.13
6 months	2	71	72	3.48	[−1.17, 8.12]	0.14
1 year	3	110	105	1.00	[−3.61, 5.61]	0.67
Supination						
3 months	4	160	158	7.73	[1.06, 10.40]	0.02*
6 months	2	71	72	9.01	[4.54, 13.49]	<0.0001*
1 year	3	110	105	1.59	[−2.91, 6.09]	0.49
Pronation						
3 months	4	160	158	1.17	[−1.95, 4.28]	0.46
6 months	2	71	72	−0.73	[−2.73, 1.26]	0.47
1 year	3	110	105	−1.11	[−3.26, 1.05]	0.32

*VLP* volar locking plate, 95 % *CI* confidence interval, *WMD* weighted mean difference

*Significant value

#### Odds ratio for total complications

**Fig. 8:**
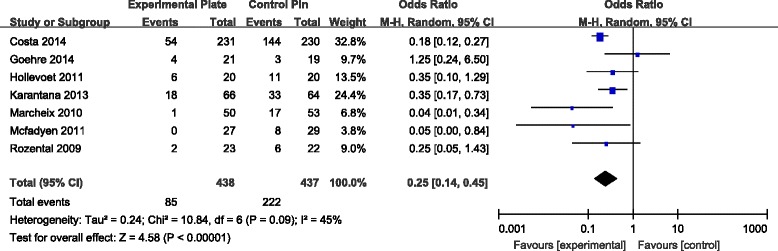
Forest plot illustrating the meta-analysis for rate of total postoperative complications

All seven RCTs [7–13] reported data on incidence rates of postsurgical complications, including superficial infection, deep infection, complex regional pain syndrome (CRPS), carpal tunnel syndrome (CTS), fracture recurrence, nerve and tendon injury, loss of reduction, additional surgery for hardware removal, pin migration and revision. Meta-analysis of overall treatment effect revealed a significantly increased risk for total complications for patients with K-wire fixation when compared to patients with VLP fixation (Fig. 8).

#### Odds ratio for specific complications

**Fig. 9:**
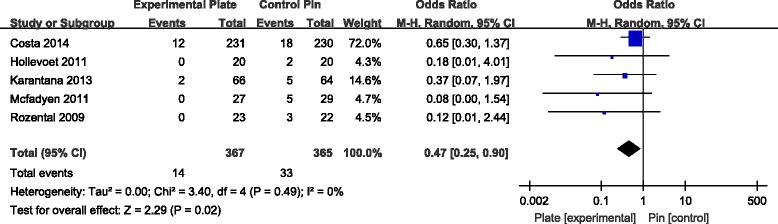
Forest plot illustrating the meta-analysis for rate of superficial infection postoperatively

The incidence of superficial infection was calculated in five RCTs [7, 9, 10, 12, 13]. The incidence of superficial infection was significantly higher for patients with K-wire fixation compared to patients with VLP fixation (Fig. 9). While the rate of CRPS, nerve injury, tenosynovitis and loss of reduction was not significantly different for the two types of fixation methods, there was a tendency for a lower total incidence of these complications in patients treated with VLP fixation (Table 3).

**Table 3:** Comparisons of postoperative complications

Complications	Number of studies	Participant		Odds ratio [95 % CI]	*p* value
		VLP	Pin		
Superficial infection	5	367	365	0.47 [0.25, 0.90]	0.02*
Deep infection	2	251	250	1.00 [0.14, 7.28]	1.00
CRPS	4	163	166	0.50 [0.17, 1.50]	0.22
CTS	4	134	132	1.13 [0.38, 3.31]	0.83
Neurological injury	4	374	376	0.72 [0.27, 1.91]	0.51
Tendon rupture	3	317	314	1.04 [0.34, 3.18]	0.94
Tenosynovitis	2	43	42	0.45 [0.06, 3.62]	0.45
Additional surgery to remove hardware	5	365	362	0.97 [0.33, 2.79]	0.95
Loss of reduction	2	48	48	0.19 [0.02, 1.76]	0.14
Total complications	7	438	437	0.25 [0.14, 0.45]	<0.00001*

*CRPS* complex regional pain syndrome, *CTS* carpal tunnel syndrome, *VLP* volar locking plate, *CI* confidence interval

*Significant value

#### Publication bias

**Fig. 10:**
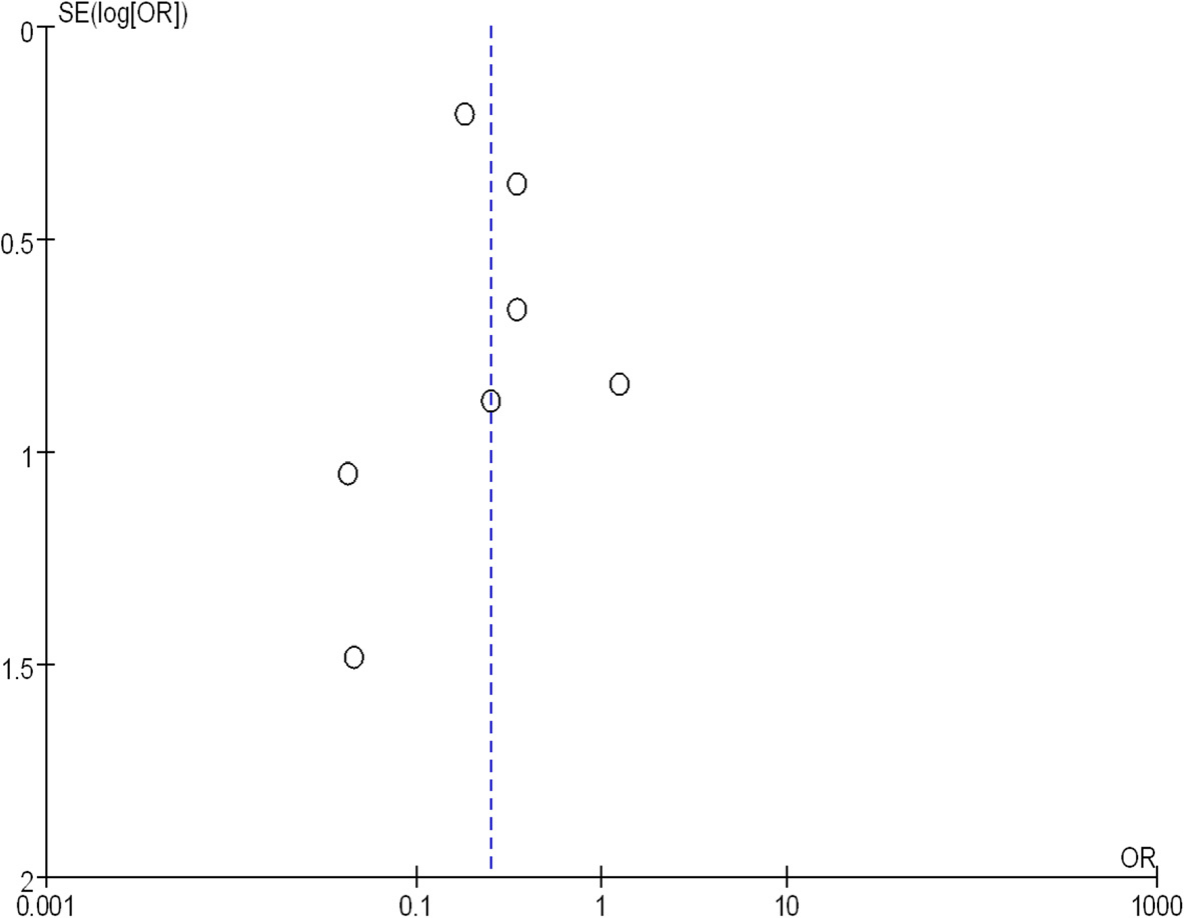
A funnel plot of the total complications to assess publication bias

A funnel plot of total complications was used to evaluate publication bias (Fig. 10). The symmetry of the funnel plot of total complications showed no evidence of bias in the small number of RCTs included. The result of Egger’s test also suggested no existence of publication bias (*P* > 0.05).

## Discussion

Prospective randomized controlled trials (RCTs) provide level I evidence [19]. Our meta-analysis pooled data from the seven RCTs identified, comparing surgical and functional outcomes of VLP and K-wire fixation techniques were compared, providing clinicians with high-quality evidence to inform their clinical decisions. Our meta-analysis showed that patients in whom DDDRF was managed using open reduction internal fixation (ORIF) with VLP had lower DASH scores, reduced total complications and lower superficial infection rates. Other specific complications were not found to be significantly different between the two fixation techniques. Grip strength and ROM of the wrist in flexion and supination were also found to be significantly better in patients with VLP, compared to those with K-wire fixation group, in the first 6 months postoperatively.

Global hand function was evaluated using the upper limb functional evaluation scoring system—the DASH score. The DASH is a validated 30-item, self-report questionnaire designed to measure physical function and symptoms in patients with musculoskeletal disorders of the upper limb, with a total score ranging between ‘0’, indicative of normal use of the upper limb, to ‘100’, indicative of a nonfunctional upper limb. It was generally agreed that the DASH scores tend to show similarity at the end of 1 year following DDDRF. No significant difference existed in DASH scores at 12 months in many recent studies [7–10, 13, 20]. However, we performed a meta-analysis of DASH scores at 3, 6 and 12 months postoperatively by pooling the data from six RCTs [7, 9–13]. DASH scores were significantly lower (i.e. less impairment in upper limb function), for all time points of measurement over 1 year postoperatively, for patients who underwent ORIF with VLP compared to patients with K-wire fixation. The difference in DASH scores between the two patient groups, however, tended to decrease over time. It is possible that the higher DASH scores for patients with K-wire fixation over the initial postoperative phase may be due to delayed onset of wrist ROM exercises. Therefore, the VLP fixation technique could be considered for patients requiring a faster return to function after injury.

Pooling the data from four RCTs [9–11, 13], our meta-analysis found that grip strength was significantly better in patients with VLP fixation at 3 and 6 months postoperatively, with no significant difference at 12 months postoperatively. We found range of wrist flexion and supination to be significantly better in patients with VLP fixation at 3 and 6 months, again with no significant difference, compared to patients with K-wire fixation, at 12 months. There were no differences for other ROMs of the wrist between the two patient groups. In our analysis, we presumed that patient-reported function and satisfaction, as recorded by the DASH, was partially related to objective assessments of wrist and hand function (i.e. ROM and grip strength) following DDDRF, which could explain statistical differences in grip strength between the two patient groups over the early postoperative period. But the ROMs of the wrist and grip strength between the two patient groups are also similar at 1 year. Even extension and pronation do not show any difference even at 6 months. The argument of improved ROMs at an earlier time is not entirely true. So the selective return of wrist movements may not benefit the patient much clinically. We did not include radiographic data in our meta-analysis because the criteria for radiographic outcomes were not uniformly reported across RCTs. Furthermore, these radiographic parameters did not necessarily correlate with subjective functional outcomes [21, 22].

Our meta-analysis also showed that VLP fixation significantly reduced the incidence of total surgical complications and superficial infections over 1 year of follow-up. In contrast, surgical complication was found to be a major problem with use of the K-wire fixation technique. The rate of superficial infections was significantly higher in patients with K-wire fixation, compared to those with VLP fixation. Once the patients developed pin track infections, the pins may also loosen, with the possibility of causing tendon irritation or attritional rupture of tendons, as well as causing irritation of the superficial branch of the radial nerve. These complications could lead to possible early removal of the pin. However, it is the common experience of most orthopaedic surgeons that smooth K-wires do not carry any significant risk of deep infection. Patients developing pin track superficial infections could require oral or intravenous antibiotics. Superficial pin track infections do not cause clinical debility in the vast majority of cases. Thus, the claim of reduced superficial infection rate may not be clinically important. Pooled data and meta-analysis showed a further, nonsignificant, tendency for lower incidence of other surgical complications in patients with VLP fixation compared to K-wire fixation, including lower risk of nerve palsy, CRPS, tendon injury and loss of reduction.

According to the result of our meta-analysis, VLP may be a superior fixation technique, over traditional K-wire fixation, for the clinical management of DDDRF. However, from a cost-analysis perspective, both Shyamalan [23] and Dzaja [24] found the cost of VLP to be two- to threefold higher than that of K-wire fixation. Surgeons must weigh all evidence when determining the optimal treatment for DDDRF in collaboration with the patient. We do champion the idea of shared decision making in orthopaedics [25]. The surgeon must provide patients with evidence-based information regarding the risks and benefits of the two surgical fixation techniques, taking into consideration a patient’s expectations, lifestyle and associated injuries in determining the most appropriate treatment approach.

Our meta-analysis has several clinical limitations. The maximum follow-up period of the RCTs was relatively short at 12 months in five RCTs [7–10, 13] and 6 months in two [11, 12]. Although an average follow-up of 12 months is sufficient to evaluate the result of DDDRF treatment [26], the results of long-term follow-up remain to be clarified. In addition, inclusion criteria were not absolutely consistent across RCTs (e.g., different fracture types and age of patients) and different levels of technical expertise of orthopaedic surgeons could also cause a certain degree of clinical heterogeneity. The current evidence is also limited by the methodological quality of the RCTs, with only two studies [10, 11] specifically stating that they followed the intention-to-treat principle. As well, although data from seven RCTs was included in the meta-analysis, the relatively small number of participants limited the statistical power of findings. Future RCTs are needed to improve the statistical outcomes.

## Conclusion

ORIF with VLP fixation provided lower DASH scores and reduced total postoperative complications, most specifically lowering the risk for postoperative superficial infection compared to K-wire fixation over a 1-year follow-up period. VLP fixation also provides better recovery of postoperative grip strength and ROM of wrist flexion and supination in the early 6-month postoperative period. However, owing to the limitation and bias of the evidence in our meta-analysis, all the above viewpoints require larger and more rigorously powered multicentre RCTs for confirmation. The only reasonable conclusion that can be drawn is that at present, there is insufficient data even on our meta-analysis to help the clinician make an informed choice.
